# Mr. M'Laren's Case of Hysteria

**Published:** 1801-03

**Authors:** G. M'Laren

**Affiliations:** Surgeon. Perth


### *To the Editors of the Medical and Phyfical Journal.*

GENTLEMEN,

IF you think the following cafe worthy inferting in your moft valuable Journal, it is at your fervice. I am, &c.

*Perth, Jan.* 19, 1801.

G. M'LAREN, Surgeon.

---

In June laft I was afked to vifit Mary Aitken, æt. 50, who lived about three miles from this town, and who, I was told, had been ill for upwards of two years, and had for the laft fix months been confined to her bed. I found her affected with violent hyfteria; fhe was of the fanguine habit, and robuft conftitution: She had taken a number of medicines commonly ufed in that difeafe, but had experienced no relief. I ordered her three dofes of mufk, one of fix, another of feven, and a third of nine grains, to be taken the three following mornings, beginning with the fmalleft. Every morning, about half an hour after fhe had taken the powder, a violent paroxyfm of the difeafe came on, throwing the body into various convulfive motions, and which having continued for near three quarters of an hour, fhe became quiet, and a copious perfpiration was produced over the whole body. I faw her again the day after fhe had ufed the three powders, when fhe found herfelf greatly relieved, but complained of being very weak; however, by the adminiftration of a few tonics, fhe found herfelf fo well in twelve days from the time I firft faw her, that fhe walked into town, and has had no return of her complaint.

